# Effect of a Novel Microwave-Assisted Induction Heating (MAIH) Technology on the Quality of Prepackaged Asian Hard Clam (*Meretrix lusoria*)

**DOI:** 10.3390/foods10102299

**Published:** 2021-09-28

**Authors:** Yi-Chen Lee, Chung-Saint Lin, Wei-Han Zeng, Chiu-Chu Hwang, Kuohsun Chiu, Tsung-Yin Ou, Tien-Hsiang Chang, Yung-Hsiang Tsai

**Affiliations:** 1 Department of Seafood Science, National Kaohsiung University of Science and Technology, Kaohsiung 811213, Taiwan; lionlee@nkust.edu.tw (Y.-C.L.); a98992011@gmail.com (W.-H.Z.); 2 Department of Food Science, Yuanpei University of Medical Technology, Hsin-Chu 30015, Taiwan; chungsl@mail.ypu.edu.tw; 3 Department of Hospitality Management, Yu Da University of Science and Technology, Miaoli 361027, Taiwan; 4 Department of Aquaculture, National Kaohsiung University of Science and Technology, Kaohsiung 811213, Taiwan; kuohsun@nkust.edu.tw; 5 Department of Marketing and Distribution, National Kaohsiung University of Science and Technology, Kaohsiung 811213, Taiwan; outy@nkust.edu.tw; 6 Department of Intelligent Commerce, National Kaohsiung University of Science and Technology, Kaohsiung 811213, Taiwan; thchang@nkust.edu.tw

**Keywords:** hard clam, microwave-assisted induction heating, microwave, quality, thermal technology

## Abstract

The microwave-assisted induction heating (MAIH) method—an emerging thermal technique—was studied to heat the prepackaged raw hard clam (*Meretrix lusoria*). The cooking effects on microbial and physiochemical qualities of clam were investigated. After the heating of the clam meat samples, the aerobic plate count (APC), psychrotrophic bacteria count (PBC), and total volatile basic nitrogen (TVBN) levels decreased with increasing heating time, but the shucking ratio, area shrinkage, and texture (hardness, cohesiveness, and chewiness) increased. In addition, the L* (lightness) and *W* (whiteness) of the clam meat samples increased significantly at the beginning of the heating period, whereas they decreased significantly with extended heating time. However, a* (redness) had the opposite trend. This study found that when clams were heated for more than 120 s at 130 °C or 150 s at 90 °C, they displayed obvious shrinking and a yellow-brown appearance, indicating that they are overcooked. After heating by MAIH for at least 110 s at 130 °C or 130 s at 90 °C, the samples were cooked well and gains a completely shucking, along with no microbial count detected. Therefore, the results indicated that the optimum heating conditions for prepackaged hard clams subjected to an MAIH machine were 130 °C for 110 s or 90 °C for 130 s.

## 1. Introduction

Microwave (MW) heating has some merits, such as reduced processing time, a fast heating rate, and minimized destruction of food nutrients and flavors [1]. MW heating has seen popular use in the food processing field, such as cooking, blanching, drying, thawing and tempering, pasteurization, and sterilization of foods [2,3]. In conventional heating techniques, heat is usually transferred from the surface to the interior, resulting in a slow heating rate, whereas in microwave heating techniques, heating occurs throughout the whole food, volumetrically and simultaneously. Therefore, microwave heating is 3–5 times faster than conventional heating [3]. Nevertheless, the non-uniformity of temperature distribution is a primary disadvantage of MW heating, which can often create cold and/or hot spots in the food [4]. The coldest location in food by MW heating can result in serious food safety concerns [5,6]. Thus, MW combined with other conventional heating methods can effectively overcome the drawbacks of the MW technique [7]. This combination known as microwave-assisted (MA) processing technology, such as MA vacuum frying, MA oven drying, MA freeze drying, and MA steam heating, have been widely applied to food processing [7]. Recently, an emerging microwave heating process, namely microwave-assisted thermal sterilization (MATS) or microwave-assisted pasteurization system (MAPS), was developed at the research group in Washington State University [8]. In MATS and MAPS, the food prepackaged in polymer containers is heated in pressurized hot water and microwave heating set at 915 MHz of frequency to perform rapid sterilization or pasteurization. In particular, the utilization of heating water as an intermediate can reduce edge heating in food packages by using MW heating [9]. This process is an effective technique to eliminate microbial count, accompanied by reducing the decomposition of nutrition and flavors in soft bag food [8]. MATS is a system consisting of four parts, including preheating, microwave heating, heat holding, and cooling, with each part having a circulatory system of water which is made up of a pressurized reservoir and heat exchanger [9]. MATS has been used to study thermal treatments for various foods, including asparagus, macaroni and cheese, salmon, chicken, mashed potatoes, and sliced beef [4,8].

**Figure 1:**
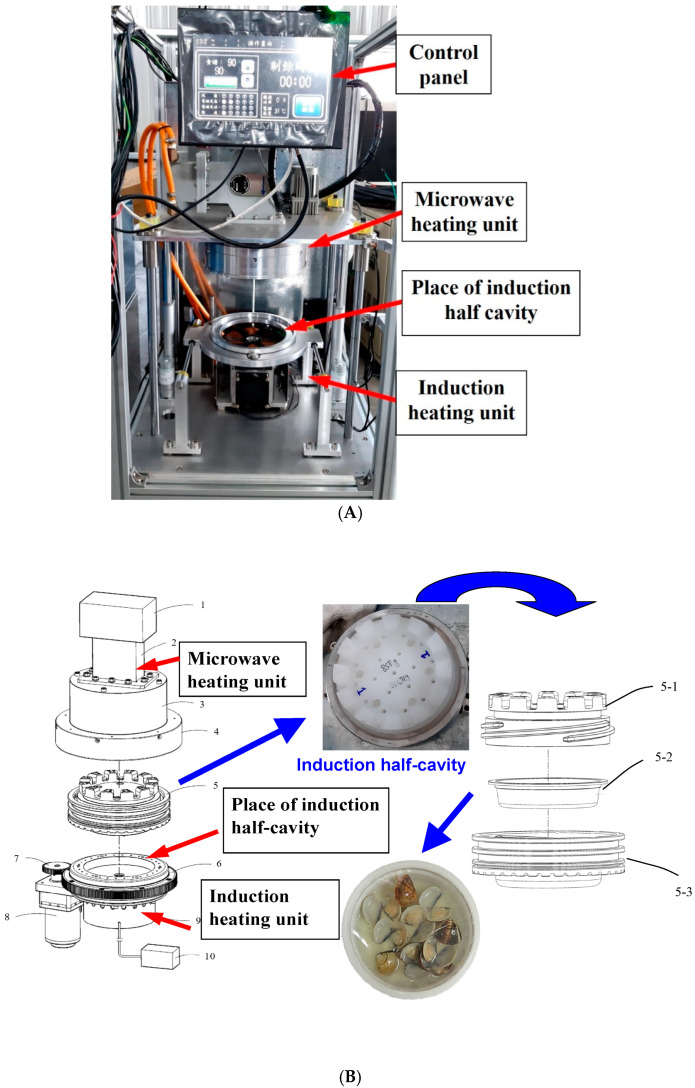
A MAIH system developed and installed at Bottle Top Machinery Co., Ltd., Nantou, Taiwan: (**A**) equipment view; (**B**) configuration of the system; 1: microwave heating unit; 2: waveguide; 3: microwave half-cavity body; 4: microwave half-cavity cover; 5: induction half-cavity; 6: revolving take-over turntable; 7: rotating gears; 8: rotating motor; 9: induction heating unit; 10: induction heating power controller; 5-1: induction half-cavity upper cover; 5-2: sealing CPET container; 5-3: induction half-cavity body.

Inspired by the work of Washington State University, a novel microwave-assisted induction heating system (MAIH) for rapid heating of prepackaged food was developed by Bottle Top Machinery, Co. in Taiwan [10]. In detail, an MAIH heating unit consists of two heating sources: a 2450 MHz microwave in the top part of machine and an electromagnetic induction heating module in the bottom part of machine (Figure 1). Before heating, the raw food ingredients are put into a heat-resistant container and then sealed with a polyethylene terephthalate (PET) film. Subsequently, the container is set in the induction half-cavity, which consists of top and bottom chambers with a tight fitting, and then it is heated by the MAIH system [10]. The rigid induction half-cavity formed between the top half microwave heating cap and bottom half induction base prevents expansion of the packaged foods when heated above 100 °C, thereby eliminating the need for overpressure, which is commonly produced by pressurized steam or water in conventional canning systems [10]. Recently, MAIH has been used for the cooking and pasteurization of shrimp for the first time in our research group [11]. Additionally, a previous piece of research also reported that after shrimps were processed by MAIH heating, the temperatures of all samples in the CPET container were within the range of 90–100 °C, as measured by thermal images; the uniform temperature distribution indicated that these heated shrimps underwent uniform heating [12].

Asian hard clam (*Meretrix lusoria*) is classified as an euryhaline bivalve mollusk and ranks second in aquaculture in Taiwan. Its production in Taiwan is approximately 40,000–50,000 metric tons each year [13]. Hard clam is an excellent source of several nutrients, including peptides, proteins, enzymes, polysaccharides, minerals, essential vitamins, essential amino acids, and apoptotic-inducing epidioxysterols, all of which could be beneficial for the health of consumers [13,14]. Therefore, hard clam essence (hot water extract) has been sold in Taiwan markets as a nutraceutical [15]. Most hard clam is consumed in cooked form, largely by boiling in soup. Heat treatment effectively inactivates some pathogens and spoilage bacteria, thereby maintaining the quality of the bivalve clam [16]. However, bivalves are filter-feeding shellfish that have the ability to bio-accumulate food pathogens and human intestinal viruses via filtering contaminated estuarine waters. When undercooked bivalves using conventional cooking methods are consumed by humans, they can cause microbiological food poisoning. Thus, the novel and uniform heating method of MAIH can be an alternative to solve this problem.

Nevertheless, no information regarding the use of MAIH for heating prepackaged hard clam exists. Therefore, the objective of this study was to use small scale MAIH for prepackaged hard clam heating, immediately after which the quality attributes of the product, including the appearance, color, texture, and microbiological and chemical quality, were observed. This MAIH research can provide a potential alternative for developing prepackaged thermal processing for the seafood industry.

## 2. Materials and Methods

### 2.1. Sample Collection and Treatment

Live hard clams (*M. lusoria*) with a body weight of 10–13 g per clam were collected from a seafood market of Kaohsiung in southern Taiwan. The clams were kept in crushed ice and transported to the Seafood Safety Laboratory, National Kaohsiung University of Science and Technology, within 1 h. After arrival, these clams were washed with clean water and then immersed in 2% salt water at room temperature and allowed to stand for depuration. Subsequently, the 10 specimens of clam (approximately 100 g) were each placed in crystallized polyethylene terephthalate (CPET) containers (13 cm internal diameter × 3.0 cm height) containing 1% saline equal to the clam weight. Afterward, the containers were sealed using a PET film and then subjected to the MAIH treatment.

### 2.2. MAIH Processing

The MAIH system comprised two units, with MW in the top half module and induction heating (IH) in the bottom half module. Before heating, the induction half-cavity containing the clam CPET container was put on the place of induction half-cavity over the induction heating unit (Figure 1B). The MW heating unit had a power of 1300 W and frequency of 2450 MHz, and the electromagnetic induction heating unit had two heating temperatures of 130 °C and 90 °C and a power of 1800 W. The MAIH heating conditions comprised two heating stages, i.e., MW combined with induction heating in the first stage (temperature rising time) and induction heating alone (without microwave heating) in the second stage (holding time) for 30 s (Table 1). At 130 °C, the total heating time was set as 80, 90, 100, 110, and 120 s; at 90 °C, the total heating time was set as 90, 110, 130, 150, and 170 s. When the heating was finished, the induction half-cavity was taken out from the MAIH equipment and immersed in ice water for 7 min. After cooling, the CPET container was removed from the induction half-cavity and then the measurements shown below were determined for the clam samples in the container.

The MAIH system is designed to provide a cavity-separable and modular composite microwave heating system which is operated at atmospheric pressure without additional pressurized steam or water (Figure 1). The cavity-separable and modular composite is designed to separate up and down, so it can flexibly adjust the power and heating time and holding time [10,12]. In addition, the MAIH system possesses some other merits, including the following: a higher heating temperature, high-efficiency induction heating transmitted to the heated object, an atmospheric pressure chamber, temperature uniformity achieved by combining microwave and induction heating, and a self-rotating function in the cavity [10,12].

### 2.3. Appearance, Shucking Ratio, and Area Shrinkage Measurements

After heating, the appearance of each CPET container with clams and shucked clam samples was photographed on a white plate using an SLR camera (EOS 60D, Canon Inc., Tokyo, Japan). Each CPET container was examined and recorded for shucking ratio, while three containers (30 specimens of clam) were taken for each heating time. After heat treatment, a shucking clam was defined as a clam with the full release of the adductor muscle. The shucking ratio (%) was calculated as follows: shucking ratio = (number of clams with full release of adductor muscle/a total of 30 clams) × 100. For area shrinkage, the area of shucked clam meat images were recorded and measured using ImageJ software Version 1.47a (National Institute of Health, Bethesda, MD, USA). The area shrinkage was calculated as follows: area shrinkage = (area of raw sample − area of heated sample/area of raw sample) × 100 [16].

### 2.4. Microbiological Analyses

With regard to APC, each 10.0 g sample of clam meat was weighed and placed in a sterilized bottle (containing 90 mL of 0.85% sterile saline) for homogenizing at a speed of 1200 rpm for 120 s [17]. Then, 1.0 mL of the stock solution was added into a sterilized test tube containing 9 mL of 0.85% sterile saline for serial dilution. Afterward, 0.1 mL taken from each dilution was spread on a trypticase soy agar (TSA, Difco, BD, Sparks, MD, USA) culture medium and incubated at 35 °C for 24–48 h, and the number of colonies on the culture dish was calculated.

To determine the psychrotrophic bacteria count (PBC), 0.1 mL of the clam homogenate solution and a 10-fold serial dilution solution prepared during the abovementioned APC determination was spread on TSA culture medium and incubated at 7 °C for 10 days. Then, the number of colonies on the culture dish was calculated as the PBC [18]. To determine the H_2_S-producing bacteria count (HBC), 0.1 mL of the clam homogenate solution and a 10-fold serial dilution solution prepared during the abovementioned APC determination was spread on triple sugar iron agar (TSIA, Difco, BD, Sparks, MD, USA) culture medium and incubated at 25 °C for 3 days. Then, the number of black colonies on the culture dish was calculated as the HBC [17]. For the determination of coliforms and *Escherichia coli*, 1.0 mL of the clam homogenate solution and a 10-fold serial dilution solution prepared during the abovementioned APC determination was transferred onto Petrifilm EC count plates (3 M Microbiology, St. Paul, MN, USA). After incubating at 35 °C for 24 h, the colonies were enumerated according to the manufacturer instructions.

### 2.5. Color Analysis

A colorimeter (CR-300 Chroma meter, Konica Minolta, Inc., Tokyo, Japan) was used to analyze the color changes of the MAIH-treated groups of clam meat. The colorimeter was first calibrated against standard white (L* = 96.72, a* = 0.11, b* = −0.14) and red plates (L* = 51.13, a* = 50.00, b* = 24.03), and then the clam meat samples were placed on a white ceramic plate. The values displayed on the screen were L* (lightness), a* (+a, red, −a, green), and b* (+b, yellow; −b, blue). Each piece of clam meat was measured three times, and the *W* (whiteness) values were calculated according to the equation below. The results were expressed as mean values for three individual clams [16]. The *W* value was calculated as follows:

(1) $$W=100-\sqrt{{\left(100-L*\right)}^{2}+a{*}^{2}+b{*}^{2}}$$

### 2.6. Texture Determination

A texture profile analysis (TPA) was conducted using a TA.XT2 Texture Analyzer (Stable Micro System Ltd., Godalming, UK) to measure textural attributes (hardness, cohesiveness, springiness, and chewiness) of hard clam. Hardness (g) refers to the amount of force exhibited in the first bite at maximum compression. Cohesiveness describes the internal adhesion force of the food. In this case, the cohesiveness is defined as the ratio between the first compression area and the second compression area. Springiness (mm) is used to characterize the restoration ratio in height or volume of a deformed sample after the deformation load is removed (i.e., the sample is subjected to the same initial condition before the load is applied). Chewiness is the energy (mJ) required to chew solid food until it can be swallowed. Before TPA measurement, the raw and heated samples were allowed to equilibrate to room temperature (approximately 22 °C), which took approximately half an hour. The following conditions were used for determination: a probe with TA39 cylinder, 2 mm D, 20 mm L; target value, 5.00 mm; predicted speed, 1.5 mm/s; test speed and return speed, 1.3 mm/s; load at trigger point, 2 g; compression rate, 50% of the original height; pressing depth, 0.5 cm; holding time, 3 s. In the TPA test, three readings were taken for each clam meat sample at three different locations, triplicate clams (*n* = 3) were tested for each heating time, and their mean levels were recorded.

### 2.7. Analysis of Total Volatile Basic Nitrogen (TVBN) and pH Value

The micro-diffusion method of Conway’s dish was used to determine the TVBN values for the clam meat samples [19]. The clam meat samples (5 g) were each placed in a 50 mL centrifuge tube containing 20 mL of 6% trichloroacetic acid (TCA) and were homogenized in a blender at 1200 rpm speed for 2 min. The homogenized solution was centrifuged at 3000× *g* for 5 min (4 °C) and filtered through Whatman No. 1 filter paper. The above steps were repeated twice. The filtrate was collected and constituted to 50 mL with 6% TCA (i.e., TCA extract) for the analysis of TVBN content. One milliliter of filtrate and 1.0 mL of saturated K_2_CO_3_ solution were transferred into the outer ring of the dish, and 1 mL of boric acid was then added into its inner ring. After the dish was placed in an incubator at 37 °C for 90 min, the TVBN absorbed by the boric acid solution in the inner ring was titrated with 0.02 N HCl. The TVBN value was presented as mg/100 g of clam sample. In terms of pH measurement, 5 g samples were added to 20 mL of deionized water to blend using a blender (FastPrep-24, MP Biomedicals; Solon, OH, USA) for 2 min at 220 rpm. A PB-10 pH meter (Satorius, Gottingen, Germany) was used to determine the pH value of this mixture.

### 2.8. Statistical Analysis

A SPSS package version 22.0 for windows (SPSS Inc., Chicago, IL, USA) was used for statistical analysis. The statistics were performed using one-way analysis of variance (ANOVA). Duncan’s multiple range test was used to determine data comparison of means in triplicates. A *p* value of less than 0.05 was regarded as statistically significant.

## 3. Results and Discussion

### 3.1. Appearance, Shucking Ratio, and Area Shrinkage of Hard Clam

**Figure 2:**
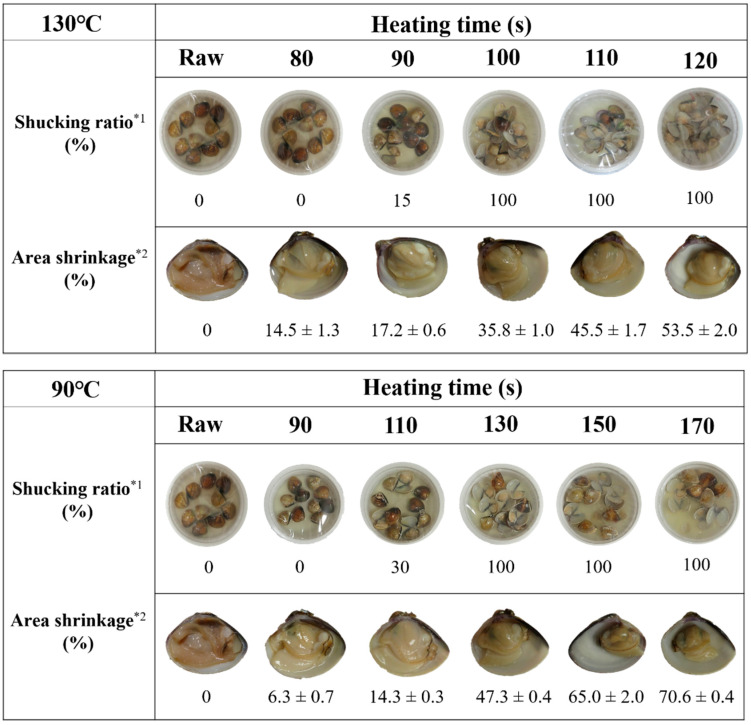
Changes in appearance, shucking ratio, and area shrinkage in hard clam at 130 °C and 90 °C for various heating times using MAIH system. *^1,^ 30 clams were used for each treatment; *^2,^ Each value is the mean ± standard deviation (*n* = 3).

Figure 2 presents the appearance, shucking ratio, and area shrinkage of hard clam processed by MAIH. At the beginning of the MAIH heating period (130 °C for 80 and 90 s, or 90 °C for 90 and 110 s), the heated clam meat had a bright and glossy appearance compared to the raw clam meat. With extended heating time, however, a dark appearance was observed in the clam meat; when clams were heated for more than 120 s at 130 °C or 150 s at 90 °C, they displayed an obvious yellow-brown color (Figure 2). In terms of the shucking effect, a longer heating time was more effective for detaching the adductor muscle. After heating at 130 °C for at least 100 s or 90 °C for at least 130 s, a 100% shucking ratio was observed (Figure 2). The reason why heat treatment could result in bivalve shucking was that high temperatures destroyed the interaction of non-covalent bonds in the tertiary structure of proteins, which led to the denaturation of muscle proteins and connective tissues, and finally caused the release of the adductor muscle to achieve the effect of shucking [16].

In addition, area shrinkage of the clam meat increased with increasing heating time. The area shrinkage after MAIH heating at 130 °C increased from 14.5% in the 80 s sample to 53.5% in the 120 s sample, while that of 90 °C heating increased from 6.3% in the 90 s sample to 70.6% in the 170 s sample (Figure 2). The result from this study is similar to the previous research conducted by Ovissipour et al. [16], who indicated that after heating whole mussels at 90 °C, area shrinkage increased with increasing time by up to 70%. This is due to the denaturation of mussel myosin and protein and shrinkage of muscle fiber diameter and sarcomere length as a result of heating [16].

### 3.2. Microbial Count Determination of Hard Clam

The microbial counts in the hard clams heated by MAIH are presented in Table 2. The initial APC and PBC of raw clam were 5.40 log CFU/g and 5.13 log CFU/g, respectively. Under heating at 130 °C, the APC and PBC levels decreased significantly with increasing heating time, and after heating for 110 and 120 s, APC and PBC were undetected (<2.0 log CFU/g). Similar to 130 °C, the APC and PBC levels decreased significantly with increasing heating time under heating at 90 °C, and at heating times above 130 s, none of the samples contained APC and PBC (<2.0 log CFU/g) (Table 2). This suggests that longer heating times by MAIH at 130 °C or 90 °C are more effective in reducing the APC and PBC in hard clam. APC and PBC are both important indexes for evaluating the quality of fresh and low-temperature-stored aquatic products. A similar finding was previously demonstrated by Lee et al. [11], who found that MAIH at 130 °C for 80 s or 90 °C for 100 s significantly reduced the APC level of white shrimp to become negligible (<2.0 log CFU/g).

Regarding the HBC and coliform counts, the raw hard clam had an initial HBC of 3.95 log CFU/g and an initial coliform of 2.72 log CFU/g (Table 2). After the hard clam samples were heated by MAIH at 130 °C for 80–120 s or 90 °C for 90–170 s, no HBC (<2.0 log CFU/g) or coliform (<1.0 log CFU/g) were found in any of the MAIH-heated samples. *E. coli* was not detected in any of the raw or heated clam samples (Table 2). This is indicates that MAIH at 130 °C or 90 °C for 80 s or 90 s, respectively, is at least effective in reducing HBC and coliform counts in hard clam. This result is similar to our previous findings, in which no HBC (<2.0 log CFU/g) or coliform (<1.0 log CFU/g) were determined in the white shrimp samples processed by MAIH at 130 °C for 80 s or 90 °C for 100 s [12]. In summary, when the heating conditions of MAIH were set at 130 °C for at least 110 s or 90 °C for at least 130 s, the MAIH technique is able to provide effective pasteurization of prepackaged hard clam.

### 3.3. Color of Hard Clam

The changes in color-related values (L*, a*, b*, and *W*) of Asian hard clams heated by MAIH are shown in Table 3. The raw clam meat had L*, a*, b*, and *W* values of 58.08, 4.27, 9.97, and 56.70, respectively. After the hard clam samples were heated by MAIH at 130 °C for 80–120 s or 90 °C for 90–170 s, the L* (lightness) and *W* (whiteness) of the clam meat increased significantly with increasing heating time at the beginning of the heating period, reaching their highest values at 130 °C for 90 s (64.89 and 62.36, respectively) and at 90 °C for 110 s (64.83 and 62.58, respectively). With extended heating time, the L* and *W* values of the samples decreased significantly to 61.11 and 58.88 at 130 °C for 120 s, respectively, and 58.14 and 54.69 at 90 °C for 170 s, respectively. However, a* (redness) had the opposite trend, which decreased with increasing heating time at the beginning of the heating period, and reached its lowest value at 130 °C for 100 s (2.15) and at 90 °C for 110 s (1.94). With extended heating time, a* increased significantly to 3.16 at 130 °C for 120 s and 3.37 at 90 °C for 170 s. In contrast, b* (yellowness) increased with increasing heating time, reaching 17.57 at 130 °C for 120 s and 17.02 at 90 °C for 170 s. In summary, the samples processed by MAIH at 130 °C for 90 s and at 90 °C for 110 s had the highest values of L* and *W*, and the lowest value of a*. Compared to the appearance results in Figure 2, they had a brighter and glossier appearance. Nevertheless, with extended heating times, the color values of the samples had the opposite trend, showing a yellower and browner color and appearance. This is due to the fact that the meat of bivalves such as oysters and clams contain a high carbohydrate content [20]. When over-heating results in an increase in the Maillard reaction between sugars and amino acids, the color of clam meat will be dark and become significantly yellowish-brown [21].

### 3.4. Texture of Hard Clam

The changes in texture properties of Asian hard clams heated by MAIH are shown in Table 4. The raw clam meat had a hardness of 31.50 (g). After the hard clam samples were heated by MAIH at 130 °C for 80–120 s or 90 °C for 90–170 s, the hardness of the clam samples increased significantly with increasing heating time, and reached its highest value at 130 °C for 120 s (61.75) and at 90 °C for 170 s (70.25). The increase in hardness of the clam samples under heating may be related to the denaturation and aggregation of myofibrillar proteins, reducing water-holding capacity and shrinking muscle fibers, and subsequently leading to a harder and more compact tissue texture [22]. The cohesiveness increased from 0.28 in the raw clam to 0.79 at 130 °C for 120 s and 0.72 at 90 °C for 170 s with increasing time (*p* < 0.05). Initially, the springiness of the raw clam was detected to be around 6.68 (mm). Although the average springiness of the heated samples decreased slightly with increasing time, there was no statistically significant difference between them and the raw samples (*p* > 0.05). Chewiness showed similar results to hardness and increased from 0.88 (mJ) in the raw clam to 2.89 (mJ) at 130 °C for 120 s and 2.98 (mJ) at 90 °C for 170 s (*p* < 0.05). Collectively, the average values of hardness, cohesiveness, and chewiness of the heated clams increased with increasing heating time. This result is similar to our previous report, in that the hardness, cohesiveness, and chewiness of white shrimps all tend to increase with increasing heating time after using the MAIH processing method [11]. The reason for the changes in tissue texture is the denaturation and aggregation of the muscle proteins at high temperatures, which causes tissue structure shrinkage [16,22].

### 3.5. TVBN and pH Values of Hard Clam

Table 5 shows the TVBN and pH values of hard clam samples processed by MAIH. TVBN consists of ammonia and other amines, such as dimethylamine (DMA) and trimethylamine (TMA) [23]. The value of TVBN is often used as an indicator of the freshness of aquatic products. The initial TVBN of the raw hard clam samples was approximately 9.24 mg/100 g. The TVBN of the clam samples processed by MAIH heating methods (130 °C and 90 °C) ranged from 2.71 mg/100 g to 4.95 mg/100 g, while there was no statistically significant difference between these samples heated for different heating times (*p* > 0.05). Overall, the clam meat samples heated by MAIH had a lower TVBN value than that of the raw samples (*p* < 0.05). When shellfish undergoes sufficient heating, the water in the meat evaporates, which simultaneously expels the volatile ammonia (amines) [12]. Additionally, Chouhan et al. [24] reported that the reduction of TVBN might be related to the heating causing degeneration of muscle proteins, shrinkage of tissue structure, and release of water, which leads to the loss of volatile amines with the water.

Initially, the pH value of the raw clam was detected to be 7.10. The pH of clam samples processed by MAIH at 130 °C was found to increase and ranged from 7.44 to 7.52, while there was no statistically significant difference between the samples heated for different heating times (*p* > 0.05). Similarly, after heating by MAIH at 90 °C, the pH of heated samples increased and ranged from 7.16 to 7.33, but there was no significant difference between the samples heated for different heating times (*p* > 0.05). Overall, the clam samples heated by MAIH had higher pH values than those of the raw samples (*p* < 0.05). This indicates that the heating generally led to an increase in the pH values of the meat, which may be due to decreases in the number of acidic groups in muscle proteins as the proteins unfold [25].

Overall, the optimum heating time for hard clam by MAIH will be one that produces a high shucking ratio, has no microbial count, and results in few changes in appearance and area shrinkage. The hard clam meat was cooked well and shucked after processing by MAIH at 130 °C for 110 s, or at 90 °C for 130 s. Under these heating conditions, the clam meat achieved a better appearance and had a better microbial quality. In addition, lower area shrinkage and higher texture levels were found in these samples. The MAIH equipment composed of MW and induction heating used in this work is an emerging and simple technology for prepackaged clam processing. Further research on this MAIH method is required to study the storage life of prepackaged hard clam after heating using MAIH.

## 4. Conclusions

The results from this study show that clams processed by MAIH at 130 °C for at least 110 s or at 90 °C for at least 130 s are effective in shucking and reducing microbial counts to a non-detectable level. However, longer heating times lead to a deleterious effect in which muscle shrinkage and a yellow-brown color are observed. Therefore, these findings indicate that the optimum cooking conditions for MAIH that cause minimum negative effects to hard clam appearance and shrinkage are heating at 130 °C for 110 s or at 90 °C for 130 s. In this study, MAIH, an emerging thermal technique for heating hard clam, was shown to provide some merits, i.e., a fast heating rate and the capacity to heat and pasteurize after being packed, thereby eliminating the post-pollution issue. Therefore, it has great potential for developing short-time in-package pasteurization processes in the food industry.

## Figures and Tables

**Table 1:** Heating parameters in MAIH equipment for hard clam treatment.

Temperature	First Stage Heating (s)	Second Stage Heating (s)	Total Heating Time (s)
	MW + IH	IH	
130 °C	50	30	80
	60	30	90
	70	30	100
	80	30	110
	90	30	120
90 °C	60	30	90
	80	30	110
	100	30	130
	120	30	150
	140	30	170

MW + IH: Microwave combined with induction heating; IH: Induction heating alone.

**Table 2:** Changes of aerobic plate count (APC), psychrotrophic bacteria count (PBC), H_2_S-producing bacteria count (HBC), coliform, and *E. coli* in hard clam at 130 °C and 90 °C for various heating times using MAIH system.

Temperatures	Heating Time (s)	APC (Log CFU/g)	PBC (Log CFU/g)	HBC (Log CFU/g)	Coliform (Log CFU/g)	*E. coli* (Log CFU/g)
Raw		5.40 ± 0.06 ^a^	5.13 ± 0.04 ^a^	3.95 ± 0.40	2.72 ± 0.17	<1.0
130 °C	80	3.70 ± 0.40 ^b^	3.97 ± 0.04 ^b^	<2.0	<1.0	<1.0
	90	3.78 ± 0.08 ^bc^	3.63 ± 0.13 ^c^	<2.0	<1.0	<1.0
	100	3.47 ± 0.06 ^bc^	3.11 ± 0.01 ^d^	<2.0	<1.0	<1.0
	110	<2.0 ^d^	<2.0 ^f^	<2.0	<1.0	<1.0
	120	<2.0 ^d^	<2.0 ^f^	<2.0	<1.0	<1.0
90 °C	90	3.40 ± 0.01 ^c^	3.14 ± 0.04 ^d^	<2.0	<1.0	<1.0
	110	3.36 ± 0.08 ^c^	2.86 ± 0.06 ^e^	<2.0	<1.0	<1.0
	130	<2.0 ^d^	<2.0 ^f^	<2.0	<1.0	<1.0
	150	<2.0 ^d^	<2.0 ^f^	<2.0	<1.0	<1.0
	170	<2.0 ^d^	<2.0 ^f^	<2.0	<1.0	<1.0

Each value represents the average ± standard deviation of three replicates; different lowercase letters indicate significant differences in the same column (*p* < 0.05).

**Table 3:** Values of color (L*, a*, b*, *W* value) in hard clam at 130 °C and 90 °C for various heating times using MAIH system.

Treatments	Heating Time (s)	L*	a*	b*	*W*
Raw		58.08 ± 1.10 *^d^	4.27 ± 0.04 ^a^	9.97 ± 0.21 ^f^	56.70 ± 1.10 ^e^
130 °C	80	60.77 ± 0.70 ^c^	3.95 ± 0.06 ^b^	13.16 ± 0.12 ^d^	58.44 ± 0.20 ^d^
	90	64.89 ± 0.80 ^a^	2.25 ± 0.11 ^e^	13.39 ± 0.22 ^d^	62.36 ± 0.40 ^a^
	100	63.05 ± 0.70 ^ab^	2.15 ± 0.20 ^ef^	13.55 ± 0.32 ^d^	59.73 ± 0.20 ^c^
	110	62.95 ± 0.51 ^b^	2.28 ± 0.11 ^e^	15.84 ± 0.15 ^b^	58.76 ± 0.14 ^d^
	120	61.11 ± 0.42 ^c^	3.16 ± 0.30 ^cd^	17.57 ± 0.28 ^a^	58.88 ± 0.30 ^d^
90 °C	90	61.60 ± 0.20 ^c^	2.19 ± 0.06 ^e^	12.91 ± 0.12 ^de^	59.43 ± 0.18 ^c^
	110	64.83 ± 0.31 ^a^	1.94 ± 0.22 ^f^	12.63 ± 0.32 ^e^	62.58 ± 0.80 ^a^
	130	63.69 ± 0.29 ^ab^	2.73 ± 0.12 ^d^	15.12 ± 0.11 ^c^	60.58 ± 0.20 ^b^
	150	62.69 ± 0.25 ^b^	2.63 ± 0.44 ^d^	15.29 ± 0.24 ^c^	59.59 ± 0.12 ^c^
	170	58.14 ± 0.30 ^d^	3.37 ± 0.09 ^c^	17.02 ± 0.25 ^a^	54.69 ± 0.22 ^f^

* Each value represents the average ± standard deviation of three replicates; different lowercase letters indicate significant differences in the same column (*p* < 0.05).

**Table 4:** Values of texture properties in hard clam at 130 °C and 90 °C for various heating times using MAIH system.

Temperatures	Heating Time (s)	Hardness (g)	Cohesiveness	Springiness (mm)	Chewiness (mJ)
Raw		31.50 ± 6.53 ^d^	0.28 ± 0.09 ^c^	6.68± 1.17 ^a^	0.88 ± 0.15 ^d^
130 °C	80	39.80 ± 5.30 ^c^	0.65 ± 0.09 ^b^	6.09 ± 0.87 ^a^	1.46 ± 0.44 ^bc^
	90	40.50 ± 2.96 ^c^	0.66 ± 0.06 ^b^	6.90 ± 0.46 ^a^	1.59 ± 0.61 ^bc^
	100	44.50 ± 6.23 ^c^	0.85 ± 0.10 ^a^	6.14 ± 1.35 ^a^	1.73 ± 0.39 ^bc^
	110	56.50 ± 5.33 ^bc^	0.81 ± 0.08 ^ab^	6.35 ± 0.53 ^a^	1.94 ± 0.58 ^b^
	120	61.75 ± 5.39 ^b^	0.79 ± 0.07 ^ab^	6.48 ± 1.03 ^a^	2.89 ± 0.39 ^a^
90 °C	90	51.50 ± 5.12 ^bc^	0.65 ± 0.08 ^b^	6.46 ± 0.55 ^a^	2.01 ± 0.67 ^b^
	110	51.33 ± 6.71 ^bc^	0.72 ± 0.07 ^ab^	6.19 ± 0.92 ^a^	2.37 ± 0.44 ^ab^
	130	53.33 ± 4.93 ^bc^	0.73 ± 0.06 ^ab^	6.06 ± 0.70 ^a^	2.23 ± 0.13 ^ab^
	150	60.33 ± 7.53 ^b^	0.79 ± 0.03 ^ab^	6.14 ± 0.15 ^a^	2.61 ± 0.57 ^ab^
	170	70.25 ± 8.84 ^a^	0.72± 0.05 ^ab^	6.28 ± 0.56 ^a^	2.98 ± 0.47 ^a^

Each value represents the average ± standard deviation of three replicates; different lowercase letters indicate significant differences in the same column (*p* < 0.05).

**Table 5:** Values of pH and total volatile basic nitrogen (TVBN) in hard clam at 130 °C and 90 °C for various heating times using MAIH system.

Temperatures	Heating Time (s)	pH	TVBN (mg/100 g)
Raw		7.10 ± 0.01 ^c^	9.24 ± 0.48 *^a^
130 °C	80	7.52 ± 0.05 ^a^	4.20 ± 1.96 ^b^
	90	7.49 ± 0.02 ^a^	4.95 ± 0.70 ^b^
	100	7.54 ± 0.04 ^a^	3.73 ± 1.71 ^b^
	110	7.44 ± 0.07 ^a^	3.27 ± 0.86 ^b^
	120	7.48 ± 0.03 ^a^	3.83 ± 0.16 ^b^
90 °C	90	7.33 ± 0.04 ^b^	4.11 ± 1.80 ^b^
	110	7.16 ± 0.08 ^b^	4.85 ± 3.08 ^b^
	130	7.21 ± 0.06 ^b^	3.83 ± 0.32 ^b^
	150	7.22 ± 0.02 ^b^	3.73 ± 0.86 ^b^
	170	7.28 ± 0.02 ^b^	2.71 ± 0.43 ^b^

* Each value represents the average ± standard deviation of three replicates; different lowercase letters indicate significant differences in the same column (*p* < 0.05).

## Data Availability

The data presented in this study are available on request from the corresponding author. The data are not publicly available, due to privacy and ethical reasons.

## References

[1] Guo Q., Sun D.W., Cheng J.H., Han Z. (2017). Microwave processing techniques and their recent applications in the food industry. Trends Food Sci. Technol..

[2] Chandrasekaran S., Ramanathan S., Basak T. (2013). Microwave food processing—A review. Food Res. Int..

[3] Verma D.K., Mahanti N.K., Thakur M., Chakraborty S.K., Srivastav P.P., Srivastav P.P., Verma D.K., Patel A.R., Al-Hilphy A.R. (2020). Microwave heating: Alternative thermal process technology for food application. Emerging Thermal and Nonthermal Technologies in Food Processing.

[4] Tang Z., Mikhaylenko G., Liu F., Mah J.H., Pandit R., Younce F., Tang J. (2008). Microwave sterilization of sliced beef in gravy in 7-oz trays. J. Food Eng..

[5] Vadivambal R., Jayas D.S. (2010). Non-uniform temperature distribution during microwave heating of food materials-A review. Food Bioprocess Technol..

[6] Hamoud-Agha M.M., Curet S., Simonin H., Boillereaux L. (2014). Holding time effect on microwave inactivation of *Escherichia coli* K12: Experimental and numerical investigations. J. Food Eng..

[7] Chizoba Ekezie F.G., Sun D.W., Han Z., Cheng J.H. (2017). Microwave-assisted food processing technologies for enhancing product quality and process efficiency: A review of recent developments. Trends Food Sci. Technol..

[8] Tang J. (2015). Unlocking potentials of microwaves for food safety and quality. J. Food Sci..

[9] Barbosa-Cánovas G.V., Medina-Meza I., Candoğan K., Bermúdez-Aguirre D. (2014). Advanced retorting, microwave assisted thermal sterilization (MATS), and pressure assisted thermal sterilization (PATS) to process meat products. Meat Sci..

[10] Chang H.I., Chin K.T., Yu Y.C., Hsieh J.K., Lin C.H. (2018). Cavity Detachable Modular Composite Microwave Heating System. Taiwan Patent.

[11] Lee Y.C., Lin C.Y., Wei C.I., Tung H.N., Chiu K., Tsai Y.H. (2021). Preliminary evaluation of a novel microwave-assisted induction heating (MAIH) system on white shrimp cooking. Foods.

[12] Tsai Y.H., Hwang C.C., Lin C.S., Lin C.Y., Ou T.Y., Chang T.H., Lee Y.C. (2021). Comparison of microwave-assisted induction heating system (MAIH) and individual heating methods on the quality of pre-packaged white shrimp. Innov. Food Sci. Emerg. Technol..

[13] Karnjanapratum S., Benjakul S., Kishimura H., Tsai Y.H. (2013). Chemical compositions and nutritional value of Asian hard clam (*Meretrix lusoria*) from the coast of Andaman Sea. Food Chem..

[14] Pan M.H., Huang Y.T., Chang C.I., Ho C.T., Pan B.S. (2007). Apoptotic-inducing epidioxysterols identified in hard clam (*Meretrix lusoria*). Food Chem..

[15] Tsai J.S., Chen J.K., Pan B.S. (2008). ACE-inhibitory peptides identified from the muscle protein hydrolysate of hard clam (*Meretrix lusoria*). Process Biochem..

[16] Ovissipour M., Rasco B., Tang J., Sablani S.S. (2013). Kinetics of quality changes in whole blue mussel (*Mytilus edulis*) during pasteurization. Food Res. Int..

[17] Sae-leaw T., Benjakul S., Vongkamjam K. (2018). Retardation of melanosis and quality loss of pre-cooked Pacific white shrimp using epigallocatechin gallate with the aid of ultrasound. Food Control.

[18] Cousin M.A., Jay J.M., Vasavada P.C., Vanderzand C., Splittstoesser D.F. (1992). Psychrotrophic microorganism. Compendium of Methods for the Microbiological Examination of Foods.

[19] Cobb B.F., Alaniz I., Thompson C.A. (1973). Biochemical and microbial studies on shrimp: Volatile nitrogen and amino nitrogen analysis. J. Food Sci..

[20] He H., Adams R.M., Farkas D.F., Morrissey M.T. (2002). Use of high-pressure processing for oyster shucking and shelf-life extension. J. Food Sci..

[21] Bindu J., Ravishankar C.N., Srinivasa Gopal T.K. (2007). Shelf life evaluation of a ready-to-eat black clam (*Villorita cyprinoides*) product in indigenous retort pouches. J. Food Eng..

[22] Kong F., Tang J., Rasco B., Crapo C. (2007). Kinetics of salmon quality changes during thermal processing. J. Food Eng..

[23] Gill T.A. (1990). Objective analysis of seafood quality. Food Rev. Int..

[24] Chouhan A., Kaur B.P., Rao P.S. (2015). Effect of high pressure processing and thermal treatment on quality of hilsa (*Tenualosa ilisha*) fillets during refrigerated storage. Innov. Food Sci. Emerg. Technol..

[25] Hamm R., Deatherage F. (1960). Changes in hydration, solubility and charges of muscle proteins during heating of meat. J. Food Sci..

